# Editorial: Computational intelligence for multimodal biomedical data fusion

**DOI:** 10.3389/frai.2026.1828869

**Published:** 2026-04-17

**Authors:** Moolchand Sharma, Umesh Gupta, Oana Geman

**Affiliations:** 1 Maharaja Agrasen Institute of Technology, New Delhi, India; 2 Bennett University, Greater Noida, India; 3 Chalmers and Gothenburg University, Gothenburg, Sweden; 4 Stefan cel Mare University of Suceava, Suceava, Romania

**Keywords:** AI driven disease diagnosis, biomedical signal processing, computational intelligence in healthcare, deep learning for healthcare, explainable AI in healthcare, multimodal biomedical data fusion

Machine learning and computational intelligence have rapidly advanced the field of biomedical data analysis, particularly in the context of multimodal data fusion. By integrating heterogeneous data sources such as physiological signals, medical imaging, and electronic health records, these approaches enable more comprehensive patient modeling and improved clinical decision-making. Recent studies have demonstrated the potential of combining advanced learning techniques with emerging technologies such as blockchain and graph-based modeling to enhance predictive performance and system reliability (Mayuranathan et al.; Vaida and Huang). At the same time, deep multimodal learning frameworks are increasingly being applied to complex neurological conditions, enabling early detection and progression analysis in diseases such as Alzheimer's (García-Gutiérrez et al.). These developments highlight the growing importance of intelligent data fusion in next-generation healthcare systems.

This Research Topic, *computational intelligence for multimodal biomedical data fusion*, was established to explore innovative techniques for integrating diverse biomedical data modalities. Its aim is to bring together research that advances the development of intelligent systems capable of extracting meaningful insights from complex and heterogeneous datasets. The five contributions included in this Research Topic address a wide range of applications, including cardiovascular disease prediction using blockchain-enabled ensemble learning (Mayuranathan et al.), multimodal graph neural network frameworks for healthcare data integration (Vaida and Huang), deep multimodal models for cognitive decline prediction (García-Gutiérrez et al.), fusion-driven analysis of surgical time-series data (Che et al.), and novel machine learning classifiers for biomedical classification tasks (Jumaah et al.). Despite their diversity, these works share a common goal of improving predictive analytics and clinical decision support through multimodal data integration.

Across the contributions, several key methodological themes emerge. A central focus is the use of multimodal learning frameworks that combine complementary data

sources to produce richer feature representations. Techniques such as ensemble learning, graph neural networks, and deep neural architectures are widely employed to capture complex relationships within biomedical datasets (Vaida and Huang; García-Gutiérrez et al.). In addition, fusion-driven approaches for time-series analysis demonstrate how temporal biomedical data can be effectively integrated to support surgical care and patient monitoring (Che et al.). These approaches reflect a broader shift toward data-driven healthcare systems, where intelligent fusion of multiple modalities enhances diagnostic and prognostic capabilities.

Another important theme highlighted in this Research Topic is the integration of emerging technologies to address challenges in healthcare data management. For instance, the incorporation of blockchain mechanisms in predictive models provides enhanced security, transparency, and trust in healthcare systems, particularly in distributed environments (Mayuranathan et al.). Similarly, the development of novel classifiers tailored to biomedical applications demonstrates the ongoing innovation in machine learning techniques designed to handle domain-specific challenges such as class imbalance and high-dimensional data (Jumaah et al.).

While these contributions demonstrate significant progress, several challenges remain for the effective translation of multimodal AI systems into real-world clinical settings. Biomedical data are inherently heterogeneous, varying in format, scale, temporal resolution, and quality. Integrating such diverse data sources requires robust preprocessing techniques, standardized representations, and scalable computational frameworks. Furthermore, issues related to interpretability, reliability, and clinical validation remain critical, as healthcare practitioners must be able to trust and understand model predictions before adopting them in practice (Vaida and Huang). Data privacy and security also continue to be major concerns, particularly when dealing with sensitive patient information across distributed infrastructures (Mayuranathan et al.).

These studies collectively emphasize that advancing multimodal biomedical intelligence requires not only improvements in predictive performance but also a strong focus on transparency, ethical considerations, and real-world applicability. Future research directions include the development of explainable AI models, robust multimodal representation learning techniques, and frameworks for secure and privacy-preserving data integration. In addition, interdisciplinary collaboration between computer scientists, clinicians, and healthcare practitioners will be essential to ensure that these technologies are effectively translated into clinical workflows.

## Conclusion

In summary, this Research Topic provides valuable insights into the evolving landscape of computational intelligence for multimodal biomedical data fusion. The contributions demonstrate how advanced AI-driven methodologies can enhance biomedical data analysis and support improved healthcare outcomes. We hope that this Research Topic will inspire further research and foster continued innovation in intelligent biomedical data integration.

